# Ultrasonographic Detection of Age-Related Structural Degeneration in the Heel Fat Pad

**DOI:** 10.7759/cureus.109907

**Published:** 2026-05-29

**Authors:** Luis Herrera, Luis A. Arráez-Aybar, Laura Flores, Ricardo García-Mata, Maria Juliana Perez-Miguelsanz

**Affiliations:** 1 Department of Radiology, Fundación Jiménez Díaz University Hospital, Madrid, ESP; 2 Department of Anatomy and Embryology, Faculty of Medicine, Complutense University of Madrid, Madrid, ESP; 3 Department of Surgery, General de la Defensa Hospital, Madrid, ESP; 4 Department of Computer Services, Complutense University of Madrid, Madrid, ESP

**Keywords:** aging, echogenicity, echostructure, foot biomechanics, heel fat pad, soft tissue imaging, tissue degeneration, ultrasonography

## Abstract

Background: Age-related changes in the heel fat pad (HFP) are not fully characterized beyond thickness measurements, despite their potential impact on foot biomechanics and function.

Objective: The aim of the study was to evaluate ultrasonographic structural changes in the HFP across different age groups and to determine whether qualitative ultrasonographic parameters provide a more sensitive assessment of age-related structural degeneration than thickness measurements alone.

Methods: A cross-sectional observational study was conducted, including 140 asymptomatic participants aged 18-89 years, stratified into three age groups (18-45, 46-65, and 66-89 years). Ultrasound assessment included measurements of thickness, echogenicity, echostructure, and Doppler flow. Statistical analyses were performed using one-way ANOVA, two-way ANOVA, and chi-square tests.

Results: Significant age-related differences were observed in echostructure, echogenicity, and horizontal fibrous band (HFB) integrity (p < 0.0001), characterized by progressive disruption and loss of compartmental organization, predominantly affecting the deep subcutaneous macrochamber (DSM) and fibrous septa. These alterations followed a consistent age-related pattern across groups. In contrast, thickness measurements demonstrated smaller and less consistent differences. No relevant intralesional Doppler signal was detected.

Conclusion: Qualitative ultrasonographic abnormalities of the HFP, particularly involving echostructure, echogenicity, and HFB integrity, appear to detect age-related structural degeneration more consistently than thickness measurements alone. Incorporating qualitative ultrasound assessment may improve the evaluation of structural tissue remodeling in asymptomatic individuals.

## Introduction

The heel fat pad (HFP) is a specialized fibro-adipose structure that plays a critical role in shock absorption and load distribution during gait, protecting the calcaneus from repetitive mechanical stress [1-3]. Its unique architecture, characterized by adipose chambers compartmentalized by fibrous septa, provides viscoelastic properties essential for maintaining foot biomechanics [4-6].

Aging is associated with structural changes in soft tissues that may compromise their mechanical properties and functional performance [7-10]. In the HFP, these changes are thought to reduce its capacity to dissipate mechanical loads, potentially contributing to increased vulnerability to injury and age-related foot disorders. Despite this, the structural mechanisms underlying HFP degeneration remain incompletely understood.

Previous studies have predominantly relied on thickness measurements as a surrogate marker of degeneration. However, findings are inconsistent, with reported age-related changes ranging from significant reductions to minimal or absent differences [9,11-15]. This variability suggests that thickness measurements alone may inadequately reflect the complex structural remodeling processes occurring within the HFP during aging. Consequently, qualitative ultrasonographic parameters capable of evaluating tissue organization and internal architecture may provide a more sensitive assessment of early degenerative changes.

Ultrasonography offers a non-invasive and widely accessible method for evaluating soft tissue structures, allowing not only morphological assessment but also characterization of internal tissue organization [3,16,17]. Ultrasonography has also been widely applied in different populations, including patients with metabolic conditions such as diabetes [18]. In particular, echogenicity, echostructure, and the integrity of fibrous septa may reflect the organization of adipose compartments and connective tissue architecture within the HFP, potentially providing earlier indicators of tissue degeneration than thickness measurements alone. These qualitative parameters may therefore represent sensitive indicators of early degeneration that precede measurable changes in thickness.

However, current evidence is largely derived from symptomatic populations or specific pathological conditions, including inflammatory disorders [19], limiting the understanding of physiological age-related changes. Data describing the progression of echostructural alterations in asymptomatic individuals remains scarce, and the relationship between these changes and aging has not been systematically characterized.

Therefore, the aim of this study was to characterize age-related ultrasonographic changes in the HFP in asymptomatic individuals, with particular emphasis on echostructure, echogenicity, and the integrity of fibrous septa as markers of tissue organization. We hypothesized that qualitative ultrasonographic abnormalities would demonstrate a more consistent age-related pattern than thickness measurements and, therefore, provide a more sensitive assessment of structural degeneration of the HFP.

## Materials and methods

A cross-sectional observational study was conducted between April and December 2025 at the ultrasound unit of a tertiary care center. 

A total of 798 consecutive individuals were initially screened. Participants were recruited using a non-probabilistic consecutive sampling approach. From this cohort, 140 asymptomatic participants aged 18-89 years were selected according to predefined inclusion and exclusion criteria. Participants were stratified into three age groups: 18-45 years (G1, n = 46), 46-65 years (G2, n = 47), and 66-89 years (G3, n = 47).

Inclusion criteria comprised the absence of heel pain and no history of foot pathology. Exclusion criteria included diabetes mellitus, prior surgery or trauma in the heel region, plantar fascia injections within the previous two years, and body mass index >25 kg/m², in order to minimize potential confounding factors affecting HFP structure.

A priori sample size estimation was performed using G*Power (version 3.1, Heinrich-Heine-Universität Düsseldorf, Düsseldorf, Germany). Assuming a medium effect size (f = 0.25) for one-way ANOVA, a significance level of α = 0.05, and a statistical power of 80%, the minimum required sample size was 128 participants. The final sample of 140 participants exceeded this requirement, ensuring adequate statistical power for between-group comparisons.

Ultrasound examinations were performed using a high-resolution system (Aplio 500, Toshiba Medical Systems, Japan) equipped with a 10 MHz linear transducer (PLT-1005BT).

**Figure 1:**
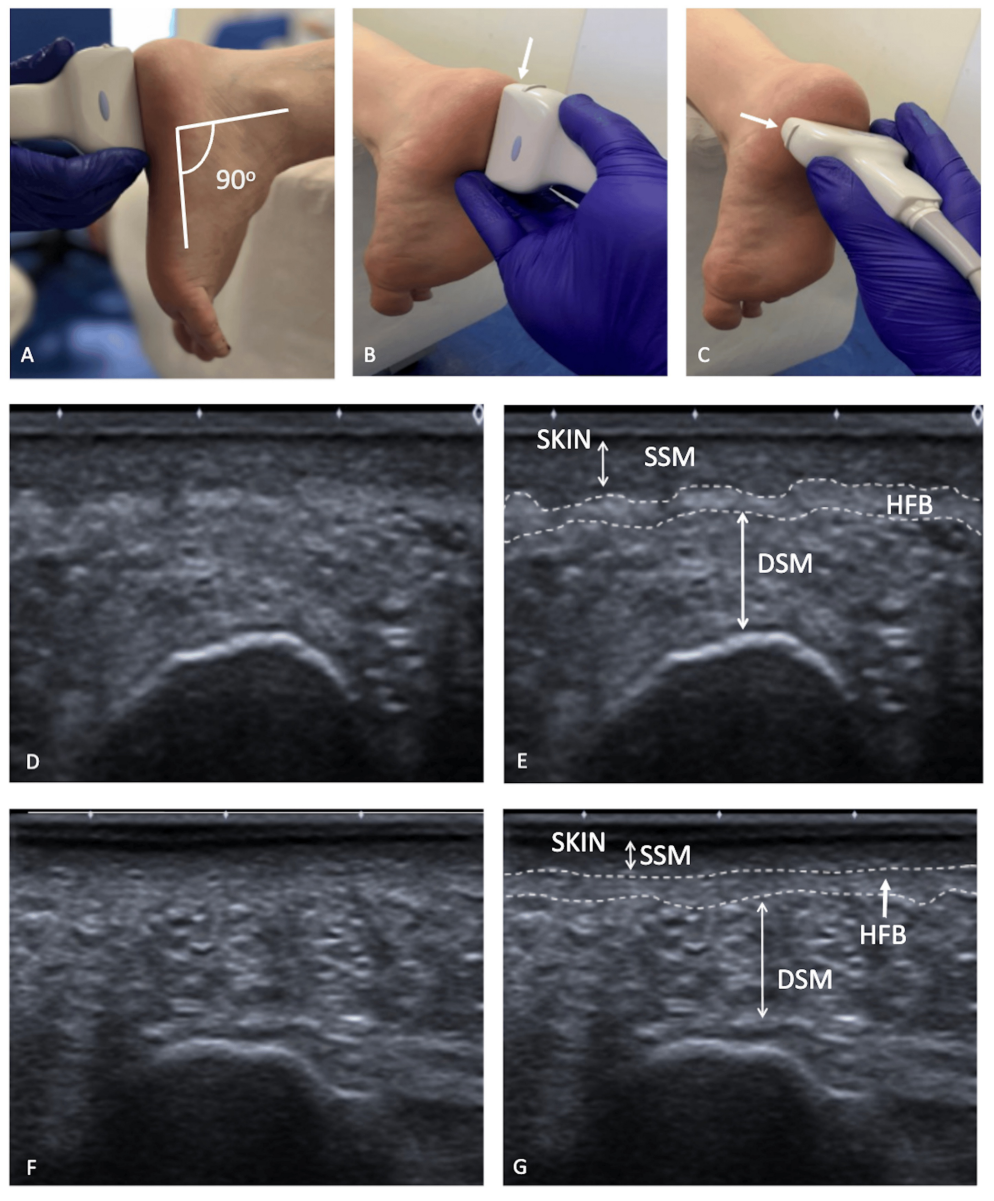
Ultrasound acquisition protocol and anatomical reference planes (A) Patient positioning in prone position with the ankle maintained at 90°. (B) Longitudinal plane acquisition with the transducer aligned parallel to the calcaneus (arrow). (C) Coronal plane acquisition with the transducer positioned perpendicular to the plantar surface (arrow). (D) Representative coronal ultrasound image of the heel fat pad (HFP). (E) Coronal image showing HFP layers. Dashed lines delimit the horizontal fibrous band (HFB), whereas vertical arrows indicate the superficial subcutaneous microchamber (SSM) and deep subcutaneous macrochamber (DSM). (F) Representative longitudinal ultrasound image. (G) Longitudinal image showing corresponding HFP layers with delimitation of the HFB (arrow) and compartment measurements indicated by vertical arrows.

All examinations were conducted under standardized conditions with participants in the prone position and the ankle maintained at 90°, ensuring consistent mechanical loading and minimizing variability in tissue thickness measurements (Figure 1).

The HFP was assessed bilaterally in longitudinal and coronal planes at the level of the plantar fascia insertion, following previously described anatomical landmarks [16]. Identical acquisition protocols were used for both feet to ensure consistency between examinations. The HFP was divided into two compartments for ultrasonographic analysis: superficial subcutaneous microchamber (SSM) and deep subcutaneous macrochamber (DSM).

Ultrasonographic parameters included total HFP thickness, compartmental thickness (SSM and DSM), echogenicity (hypoechoic, isoechoic, or hyperechoic), echostructure (organized or disorganized), integrity of the horizontal fibrous band (HFB), and color Doppler assessment for detection of intralesional or perforating vascular signals within the HFP.

Particular attention was given to the integrity of the HFB and the degree of compartmental organization. All ultrasound images were acquired independently by two experienced operators (>5 years in musculoskeletal ultrasound) using a standardized acquisition protocol.

To minimize measurement bias, transducer pressure was kept as low as possible and consistent across examinations. A pilot study was conducted in 30 subjects (not included in the final analysis) to standardize acquisition procedures and confirm the reproducibility of imaging planes.

All images were subsequently reviewed by a third independent evaluator to ensure adherence to anatomical reference points and classification criteria. Interobserver variability was minimized through protocol standardization, operator training, and independent image verification by a third reviewer. Although formal reliability statistics were not calculated, consistency was ensured through predefined acquisition and evaluation.

Echostructure was classified as organized when clear differentiation between the SSM and DSM compartments and identifiable fibrous septa were preserved. Disorganized echostructure was defined by partial or complete loss of compartmental differentiation, fragmentation or absence of fibrous septa, and heterogeneous internal architecture.

Echogenicity was assessed relative to surrounding tissues and categorized as hypoechoic, isoechoic, or hyperechoic. Echogenicity classification was based on the relative contrast between adipose compartments and surrounding fibrous septa using predefined ultrasonographic criteria.

The HFB was classified as intact when continuously visualized across the examined plane, fragmented when focal discontinuities were identified, and absent when no recognizable hyperechoic band could be visualized.

Statistical analyses were performed using SAS software (version 9.4; SAS Institute Inc., Cary, North Carolina). Continuous variables were expressed as mean ± standard deviation (SD), and categorical variables as frequencies and percentages.

Comparisons between age groups were conducted using one-way analysis of variance (ANOVA). When significant differences were detected, post hoc comparisons were performed using Duncan’s multiple range test.

The interaction between age and sex was analyzed using two-way ANOVA (type III sums of squares). Categorical variables, including echogenicity, echostructure, and HFB status, were analyzed using the chi-square test. A two-tailed p-value < 0.05 was considered statistically significant.

Because bilateral ultrasound measurements were obtained, the mean value of both feet was used for quantitative analyses in order to reduce the potential influence of within-subject laterality. Descriptive comparisons between right and left feet showed no relevant differences. The absence of mixed-effects modeling is acknowledged as a methodological limitation.

The study was approved by the local Institutional Review Board (reference EO_206-21_FJD). All procedures were conducted in accordance with the Declaration of Helsinki. Written informed consent was obtained from all participants prior to inclusion.

## Results

A total of 140 participants meeting the inclusion criteria were analyzed. Significant age-related differences were identified in echogenicity, echostructure, and HFB integrity across both feet (Tables 1, 2). Structural ultrasonographic alterations followed a progressive age-related pattern, whereas thickness measurements demonstrated comparatively smaller and less consistent differences between groups.

Echostructure and echogenicity

Significant age-group differences in echogenicity were observed across both feet (Table 1).

**Table 1:** Distribution of echogenicity patterns of the heel fat pad by age groups and foot laterality Values are expressed as n (%). Percentages are calculated within each age group. Statistical analysis was performed using the chi-square test. SSM, superficial subcutaneous microchamber; DSM, deep subcutaneous macrochamber.

Variables	G1 (18-45 years), n = 46	G2 (46-65 years), n = 47	G3 (66-89 years), n = 47	p-value
Left foot				
SSM hyperechoic	0	23 (48.9)	4 (8.5)	<0.0001
SSM hypoechoic	46 (100)	20 (42.6)	4 (8.5)	
SSM isoechoic	0	4 (8.5)	39 (83.0)	
DSM hyperechoic	46 (100)	20 (42.6)	4 (8.5)	<0.0001
DSM hypoechoic	0	23 (48.9)	4 (8.5)	
DSM isoechoic	0	4 (8.5)	39 (83.0)	
Right foot				
SSM hyperechoic	0	25 (53.2)	6 (12.8)	<0.0001
SSM hypoechoic	46 (100)	18 (38.3)	2 (4.2)	
SSM isoechoic	0	4 (8.5)	39 (83.0)	
DSM hyperechoic	46 (100)	20 (42.6)	4 (8.5)	<0.0001
DSM hypoechoic	0	23 (48.9)	4 (8.5)	
DSM isoechoic	0	4 (8.5)	39 (83.0)	

For echostructure, marked differences were observed for HFB status across both feet, whereas DSM heterogeneity reached significance only in the right foot, and SSM echostructure remained unchanged (Table 2).

**Table 2:** Distribution of echostructure patterns of the heel fat pad by age groups and foot laterality Values are expressed as n (%). Categories were not mutually exclusive for echostructural abnormalities. P-values were calculated using the chi-square test. NA: statistical comparison was not applicable because SSM echostructure remained homogeneous in all groups. SSM: superficial subcutaneous microchamber; DSM: deep subcutaneous macrochamber; HFB: horizontal fibrous band.

Variables	G1 (18-45 years), n = 46	G2 (46-65 years), n = 47	G3 (66-89 years), n = 47	p-value
Left foot				
SSM homogeneous	46 (100)	47 (100)	47 (100)	NA
DSM heterogeneous	23 (50.0)	37 (78.7)	35 (74.5)	0.08
HFB disruption	16 (30.0)	37 (78.7)	10 (21.8)	<0.0001
Right foot				
SSM homogeneous	46 (100)	47 (100)	47 (100)	NA
DSM heterogeneous	25 (54.3)	41 (87.2)	33 (70.2)	0.04
HFB disruption	17 (36.9)	41 (87.2)	10 (21.3)	<0.0001

**Figure 2:**
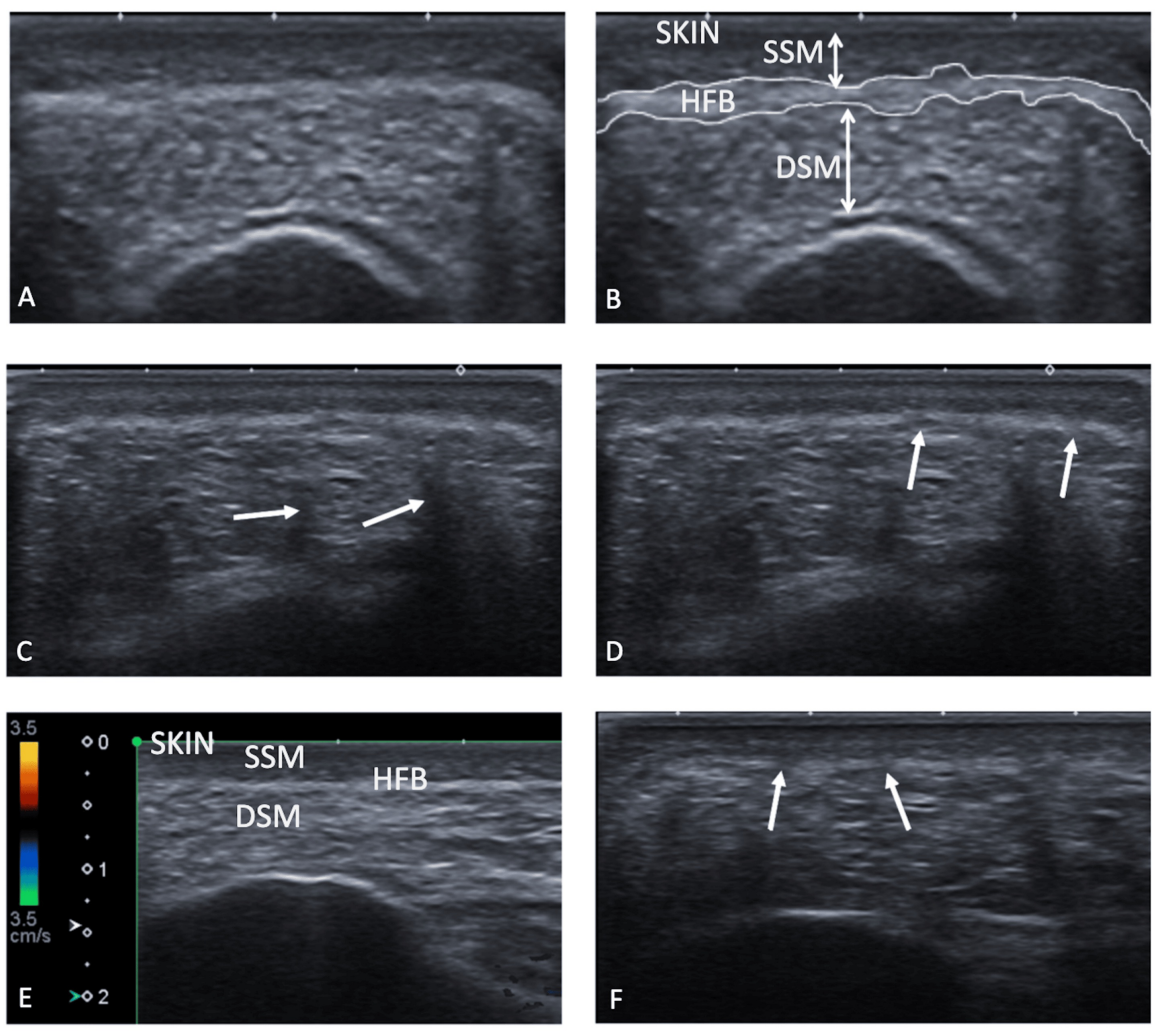
Ultrasound features of the heel fat pad in group G1 (18-45 years) (A–B) Coronal images showing preserved echostructure with clear differentiation between the SSM and DSM. The HFB appears as a hyperechoic boundary between both compartments. (C) Coronal image showing early hypoechoic grooves within the DSM (arrows). (D) Coronal image demonstrating focal disruption of the HFB (arrows). (E) Longitudinal image showing preserved compartmental organization and absence of relevant Doppler signal within the HFP. (F) Longitudinal image illustrating focal disruption of the HFB and associated echogenic alterations within the DSM (arrows). HFP: heel fat pad, HFB: horizontal fibrous band, SSM: superficial subcutaneous microchamber, DSM: deep subcutaneous macrochamber.

In G1 (18-45 years), the HFP exhibited a well-preserved echostructure, with clear differentiation between the SSM and DSM compartments and a continuous HFB (Figure 2A-B). Early focal discontinuities of the HFB were observed in individuals approaching the upper age range, accompanied by initial separation of adipose chambers (Figure 2C-D).

**Figure 3:**
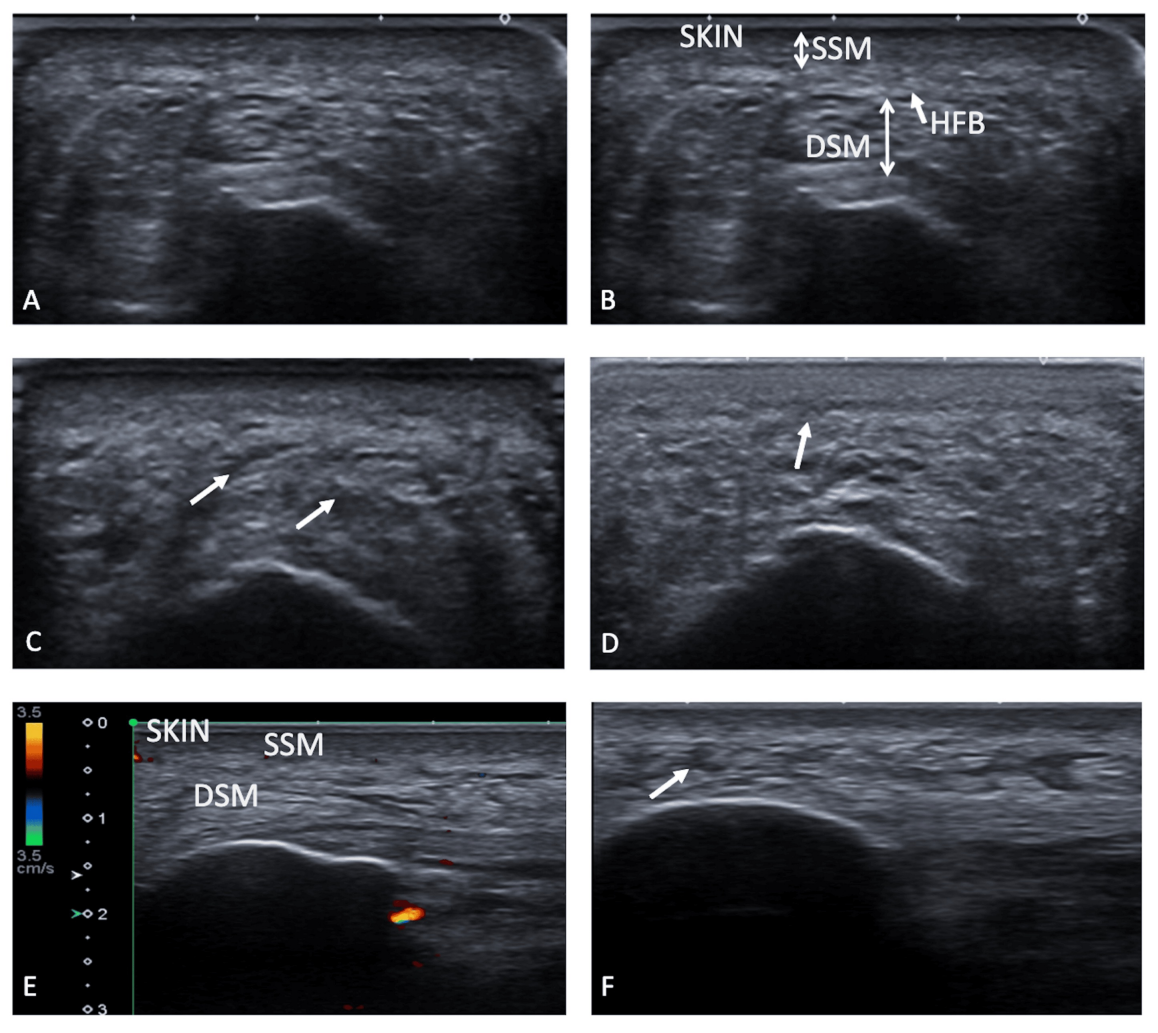
Ultrasound features of the heel fat pad in group G2 (46-65 years) (A) Representative coronal ultrasound image showing structural disorganization of the HFP. (B) Coronal image demonstrating reduced definition and fragmentation of the HFB (arrow), with identification of the SSM and DSM. (C) Coronal image showing hypoechoic sulci within the DSM (arrows). (D) Coronal image demonstrating focal disruption of the HFB (arrow). (E) Longitudinal image showing the absence of a relevant Doppler signal and reduced visualization of the HFB. (F) Longitudinal image demonstrating diffuse echostructural alteration of the DSM with hypoechoic structural changes (arrow). HFP: heel fat pad, HFB: horizontal fibrous band, SSM: superficial subcutaneous microchamber, DSM: deep subcutaneous macrochamber.

In G2 (46-65 years), a marked increase in structural disorganization was observed. HFB disruption reached its highest frequency in this group, whereas in G3, the HFB was frequently no longer distinguishable due to diffuse loss of structural differentiation. In addition, the DSM showed a heterogeneous echostructure with hypoechoic sulci (Figures 3A-D). These alterations were observed in both coronal and longitudinal planes (Figure 3E-F).

**Figure 4:**
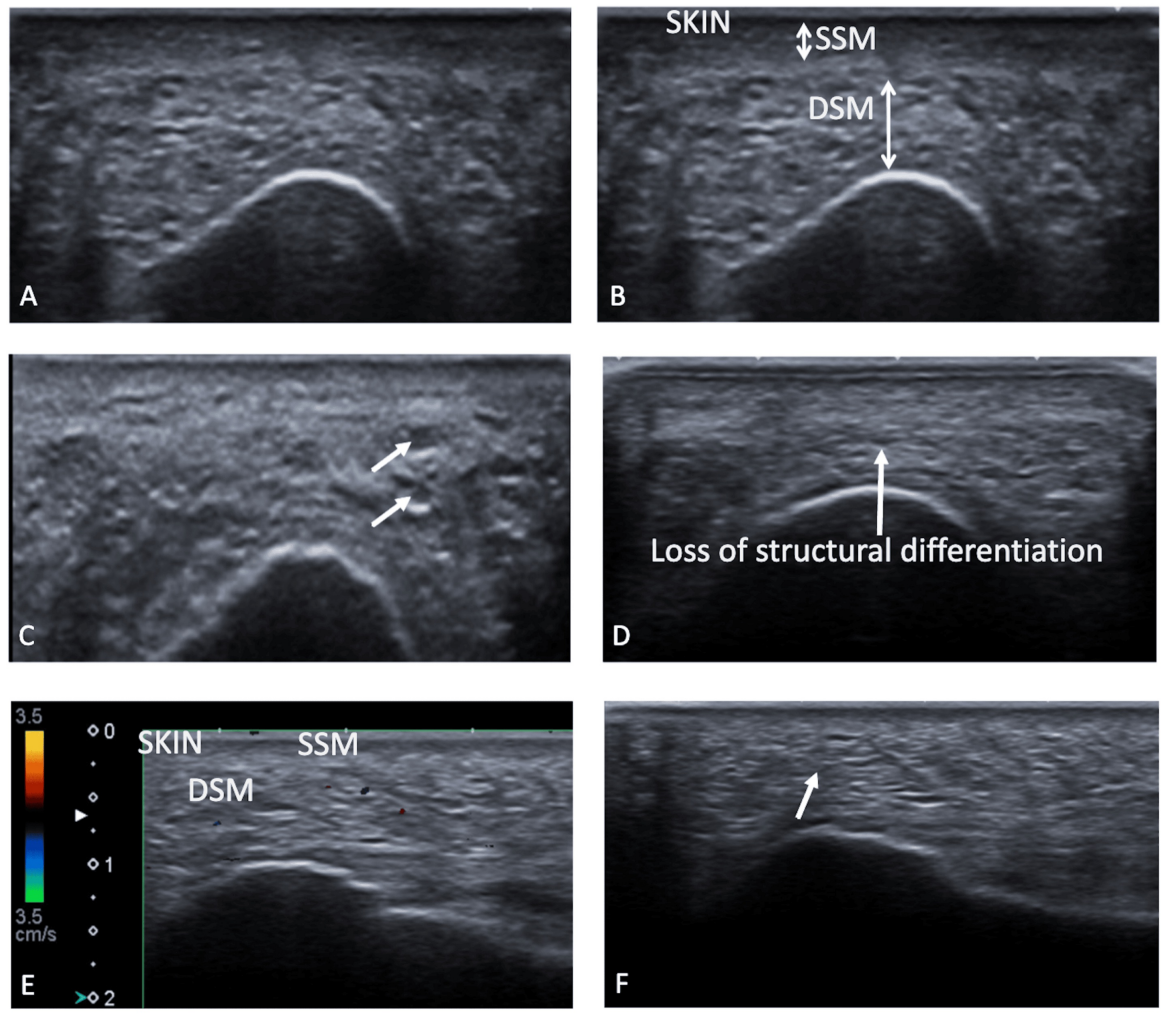
Ultrasound features of the heel fat pad in group G3 (66-89 years) (A–B) Coronal images showing homogeneous echostructure with loss of differentiation between the SSM and DSM. The HFB is no longer distinguishable. (C) Residual hypoechoic grooves may occasionally persist within the DSM (arrows). (D) Coronal image demonstrating diffuse loss of structural differentiation within the heel fat pad (arrow). (E) Longitudinal image showing the absence of a relevant Doppler signal. (F) Longitudinal image demonstrating diffuse homogeneous echostructure without clear compartmental differentiation (arrow). HFB: horizontal fibrous band, SSM: superficial subcutaneous microchamber, DSM: deep subcutaneous macrochamber.

In G3 (66-89 years), the HFP demonstrated a predominantly homogeneous echostructure, with loss of clear compartmental differentiation and near-complete absence of the HFB (Figure 4A, D, F). Compared with G2, the lower prevalence of visible HFB disruption in G3 appeared to reflect advanced architectural loss of the fibrous band itself rather than preservation of structural integrity. Residual hypoechoic grooves were occasionally identified within the DSM (Figure 4).

**Figure 5:**
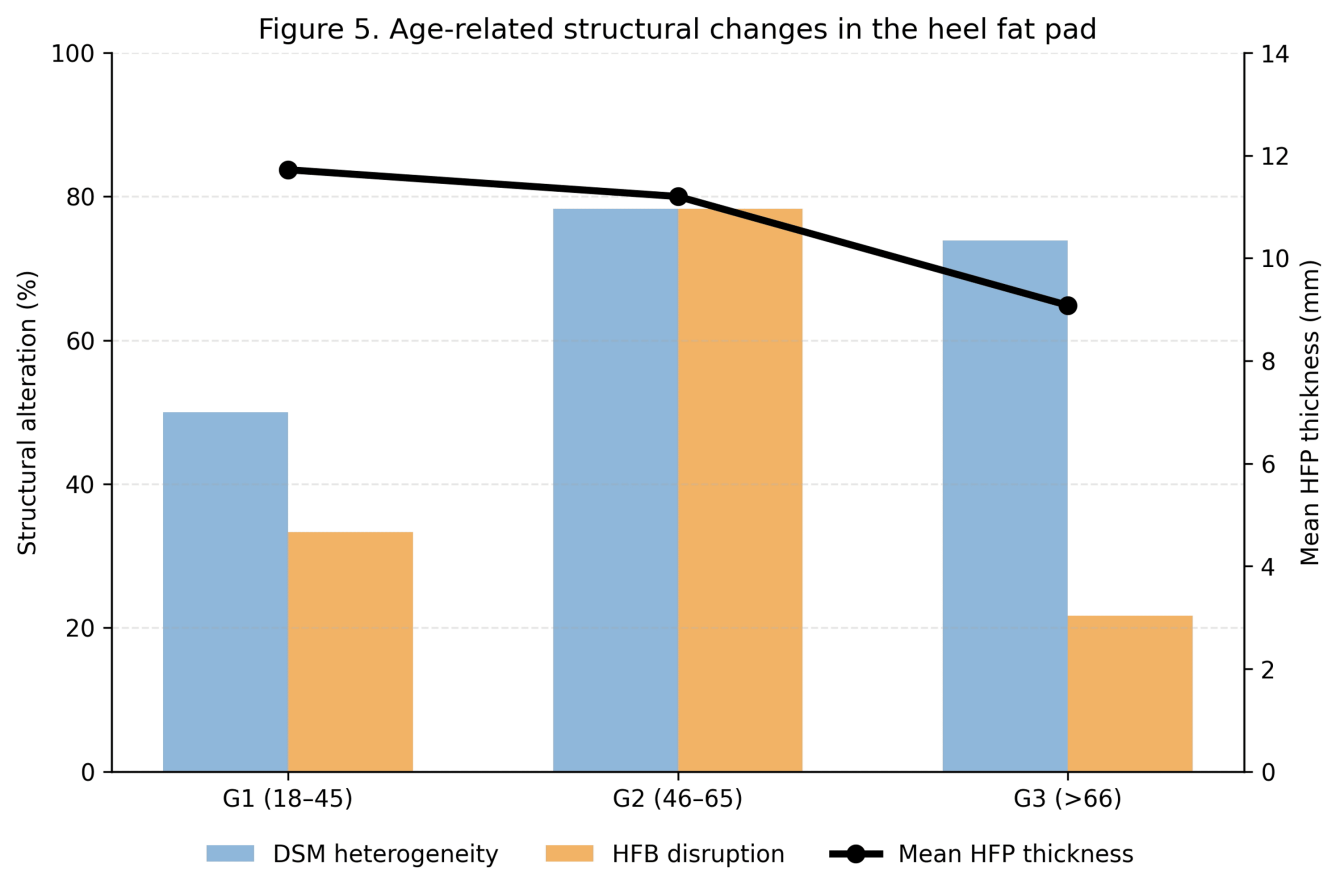
Progressive echostructural degeneration of the heel fat pad across age groups compared with relatively limited thickness variation Grouped bars show the pooled bilateral prevalence of DSM heterogeneity and visible HFB disruption across age groups (G1–G3). The reduction in visible HFB disruption in G3 likely reflects advanced architectural loss and absence of the fibrous band itself rather than preservation of structural integrity. The line represents the mean total HFP thickness (mm). Structural ultrasonographic abnormalities demonstrated a progressive age-related pattern, whereas thickness measurements showed a comparatively smaller and less consistent variation across groups. HFP: heel fat pad, HFB: horizontal fibrous band, DSM: deep subcutaneous macrochamber.

These findings are summarized in Figure 5, which illustrates the progressive increase in structural alterations across age groups in contrast to the relatively stable pattern of thickness measurements.

Echogenic contrast between compartments decreased progressively across age groups (Table 1). No relevant intralesional Doppler vascular signal was detected within the HFP in any age group, except for isolated perforating vessels observed sporadically in longitudinal views (Figures 2E, 3E, 4E).

Thickness analysis

Thickness measurements demonstrated smaller and less consistent differences across age groups when compared with the progressive alterations in echostructure, echogenicity, and HFB integrity (Table 3).

**Table 3:** Thickness measurements of the heel fat pad and plantar fascia by age groups Values are expressed as mean ± standard deviation. Different superscript letters indicate statistically significant differences between groups according to Duncan’s post hoc test. P values correspond to one-way ANOVA across age groups. SSM: superficial subcutaneous microchamber, DSM: deep subcutaneous macrochamber, HFP: heel fat pad, PF: plantar fascia.

Variable	G1 (18-45 years)	G2 (46-65 years)	G3 (66-89 years)	p-value
SSM (mm)	2.00 ± 0.42	2.02 ± 0.30	1.70 ± 0.46	0.10
DSM (mm)	8.40 ± 3.00	9.17 ± 1.44	7.83 ± 1.74	0.37
HFP (mm)	11.72 ± 2.20^ A^	11.20 ± 1.26^ A^	9.08 ± 1.15^ B^	<0.0001
PF (mm)	3.65 ± 0.93 ^A^	3.5 ± 0.39 ^A^	3.1 ± 0.55 ^B^	<0.0001

The SSM demonstrated a decreasing trend with age, reaching statistical significance in selected comparisons. In contrast, DSM thickness showed minor variations without consistent statistical significance. Total HFP thickness showed smaller and less consistent differences across groups compared with echostructural alterations. Plantar fascia thickness showed significantly lower values in G3 compared with G1 (Table 3).

Sex-related differences

No consistent sex-related differences were observed in echostructure or echogenicity patterns. In contrast, some differences were identified in thickness parameters. DSM thickness showed statistically significant differences between sexes (p = 0.01), particularly in the right foot. Total HFP thickness showed borderline significance (p = 0.05), while SSM differences were limited and inconsistent. Plantar fascia thickness did not show significant sex-related differences.

Age-sex interaction

No consistent statistically significant interaction between age and sex was identified. Descriptive trends indicated a progressive decrease in SSM thickness in men across age groups, whereas in women, SSM values remained relatively stable until late age, with a decrease observed in G3. DSM thickness showed a gradual decrease in men, while in women, a slight increase in G2 was followed by a reduction in G3. Total HFP thickness decreased progressively in men, whereas in women, minor fluctuations were observed without a clear pattern.

Laterality

No significant differences were observed between right and left feet for any of the analyzed variables. Overall, echostructural alterations followed an age-related evolutionary pattern characterized by maximal HFB disruption in G2 and diffuse architectural loss in G3, whereas thickness parameters showed limited sensitivity to these changes. Figure 5 further illustrates this dissociation between structural alterations and thickness measurements across age groups.

## Discussion

The present cross-sectional study demonstrates that age-related changes in the HFP are characterized predominantly by progressive alterations in echostructure, echogenicity, and HFB integrity, whereas thickness measurements showed comparatively smaller and less consistent differences across age groups. This dissociation, summarized in Figure 5, supports the hypothesis that qualitative ultrasonographic abnormalities may represent more sensitive markers of age-related structural degeneration than thickness measurements alone. Because of the cross-sectional design, temporal progression cannot be definitively established; however, the observed pattern is consistent with a continuum of progressive structural remodeling associated with aging.

Previous investigations evaluating age-related HFP degeneration have focused predominantly on thickness measurements, with inconsistent findings ranging from significant reductions to minimal or absent differences [7,9,11-15]. Similar variability has also been reported regarding the relationship between HFP thickness and demographic or biomechanical factors [20]. The present results are consistent with these observations, as thickness parameters demonstrated less pronounced and less consistent variation than qualitative ultrasonographic abnormalities. In contrast, progressive alterations in echogenicity and internal tissue organization were consistently identified across age groups, suggesting that structural ultrasonographic assessment may better reflect age-related remodeling processes within the HFP.

From a structural perspective, the progressive disruption of fibrous septa and loss of compartmental organization observed in older participants are consistent with degenerative remodeling processes previously described in adipose and connective tissues [10]. Notably, SSM echostructure remained relatively preserved across all age groups, whereas alterations predominantly affected the DSM and the HFB, supporting the concept that deeper compartmental structures may be more vulnerable to age-related degeneration. The HFB demonstrated progressive fragmentation in middle-aged participants, followed by reduced visualization in older individuals, likely reflecting advanced architectural loss and disappearance of recognizable fibrous boundaries rather than preservation of structural integrity. This interpretation is further supported by the increasingly homogeneous echostructural appearance observed in G3.

The transition from a well-defined compartmental organization in younger individuals to a diffuse, homogeneous echostructure in older participants may reflect advanced simplification of tissue architecture. Similar reductions in structural heterogeneity have been described in other degenerative soft tissue processes and are frequently associated with diminished functional capacity [21,22]. In this context, the progressive reduction in echogenic contrast between compartments may represent loss of differentiation between adipose chambers and fibrous septa, potentially compromising the viscoelastic shock-absorbing properties of the HFP.

Ultrasonography has been widely applied in the evaluation of pathological conditions involving the heel, including plantar fasciitis, diabetic foot disorders, and inflammatory diseases [17-19,23]. However, studies specifically characterizing physiological age-related structural remodeling in asymptomatic individuals remain limited. The present findings suggest that routine ultrasound evaluation of the heel should incorporate qualitative parameters such as echostructure, echogenicity, and HFB integrity in addition to thickness measurements alone. Standardized qualitative ultrasound assessment may therefore improve recognition of subclinical structural degeneration in aging populations before overt functional impairment becomes clinically apparent.

From a clinical perspective, these findings support the concept that structural ultrasonographic alterations may precede clinically evident symptoms and macroscopic thickness changes. Incorporating qualitative assessment into routine HFP evaluation may therefore improve early identification of age-related degeneration and refine imaging interpretation in older individuals. This may be particularly relevant given previous reports linking age-related foot structural changes with impaired mobility, altered plantar loading, and increased fall risk [24].

Several limitations should be acknowledged. First, the cross-sectional design prevents the determination of temporal causality and longitudinal progression. Second, elastography was not incorporated, precluding quantitative assessment of tissue stiffness and mechanical properties. Third, only asymptomatic participants were included, limiting direct clinical correlation with symptomatic heel disorders. In addition, formal interobserver reliability statistics such as ICC or kappa coefficients were not prospectively calculated for qualitative ultrasound classifications. Nevertheless, several measures were implemented to reduce variability, including standardized acquisition protocols, pilot testing, operator training, independent image acquisition, and third-reviewer verification. Finally, factors including physical activity, footwear, and foot morphology were not controlled and may have influenced HFP structure.

Future investigations should incorporate longitudinal designs, symptomatic populations, formal reproducibility analyses, and quantitative techniques such as elastography to better define the relationship between structural degeneration, tissue mechanics, and clinical outcomes [25].

## Conclusions

Age-related degeneration of the HFP is characterized predominantly by progressive alterations in echostructure, echogenicity, and HFB integrity, whereas thickness measurements demonstrate comparatively smaller and less consistent differences across age groups. These findings suggest that qualitative ultrasonographic abnormalities, particularly involving the DSM and fibrous septa, may represent sensitive markers of structural tissue remodeling associated with aging.

Qualitative ultrasound assessment, therefore, provides additional information beyond conventional thickness measurements and may contribute to a more comprehensive evaluation of HFP degeneration in asymptomatic individuals. Incorporating qualitative ultrasonographic parameters into routine assessment may improve recognition of early structural alterations affecting the plantar heel region.
